# A patient with epithelioid pleural mesothelioma (Myxoid variant) who survived for a long period without treatment

**DOI:** 10.1016/j.rmcr.2021.101381

**Published:** 2021-03-08

**Authors:** Keiichi Mizuhashi, Kenzo Okamoto, Takumi Kishimoto

**Affiliations:** a Center for Asbestos-Related Diseases, Toyama Rosai Hospital, Uozu-shi, Toyama, Japan; b Department of Pathology, Hokkaidoutyuuou Rosai Hospital, Iwamizawa-shi, Hokkaidou, Japan; c Research and Training Center for Asbestos-Related Diseases, Okayama-shi, Okayama, Japan

**Keywords:** Epithelioid mesothelioma, Myxoid variant, Long-term survival, No history of asbestos exposure, Female

## Abstract

Pleural mesothelioma is a disease with a very poor prognosis. Here, we report a mesothelioma patient who survived for 5 years and a half. As a result of the autopsy, the tumor was diagnosed as a myxoid variant, which is internationally proposed as a histological subtype of epithelioid mesothelioma with a relatively favorable prognosis. Since patients with this disease are expected to survive for a long period even without treatment, careful determination of the therapeutic approach is considered necessary. This report is considered to be the first of a myxoid variant epithelioid pleural mesothelioma in Japan.

## 1 Introduction

According to mortality statistics by the Ministry of Health, Labour and Welfare of Japan, the number of deaths due to mesothelioma throughout Japan in 2018 was 1,512, consisting of 1275 males and 237 females (male/female ratio = 5.38:1) [1]. Asbestos exposure is considered to have been the cause of the disease in about 80% of the patients, and pleural mesothelioma accounted for about 80% of all cases. Patients with pleural mesothelioma have a chance of cure only if diagnosed at a very early stage and undergo extrapleural pneumonectomy. In other patients, the survival period is about 1–2 years, even with a combination of cisplatin + pemetrexed, which is presently the standard chemotherapy for mesothelioma. According to the survey of the Japanese Association of Clinical Cancer Centers (compiled in February 2018) cited in the Guidelines for Malignant Mesothelioma in the Guidelines for Diagnosis and Treatment of Lung Cancer 2018, the 5-year survival rate by stage is 14.6% for stage I, 4.5% for stage II, 8.0% for stage III, and 0.0% for stage IV. The prognosis is extremely poor in all stages [2]. However, since the immune checkpoint inhibitor nivolumab began to be covered by health insurance in the summer of 2018 in Japan, some improvement in the therapeutic outcome is expected for the future. We encountered a female patient with pleural mesothelioma without a clear history of asbestos exposure who was diagnosed during a health checkup and survived for five years and six months even without treatment. Since autopsy could be performed, we report this case with details of the histopathological findings.

### 1.1 Case presentation

The patient was an 82-year-old female with dyspnea as the primary complaint. She had no particular past or familial history. She used to be a nurse (until the age of 72 years) and had no history of occupational asbestos exposure. There was also no history of residence in an asbestos-contaminated environment.

Regarding the history of the present illness, she presented with abnormal chest radiographs (Fig. 1a) during a community health screening in July 2006. Still, she left the findings unattended because of the absence of symptoms. On a retrospective review of the plain chest radiographs taken one year before, in July 2005, the right costophrenic angle was already blunted. The plain chest radiographs taken during the community health screening in July 2007 indicated abnormalities again (Fig. 1b). At this time, as the chest radiographs showed a mass in the right thoracic wall in addition to right pleural effusion, the patient was referred to a hospital for close examination. In the chest CT scans taken in July 2007 (Fig. 2), a small amount of pleural effusion was noted on the right side, and pleural masses were found in the right anterior mediastinum and anterior and lateral thoracic regions. A percutaneous pleural needle biopsy was performed for the mass of the right lateral chest wall, and a diagnosis of pleural mesothelioma was made. The pathologist in charge of the needle biopsy described the pathological findings as epithelioid mesothelioma with edematous stroma, and intracytoplasmic mucinous vacuoles are outstanding. However, when we re-evaluated the specimen, it was an epithelioid mesothelioma with abundant myxoid stroma and was completely homogeneous with the histological image at autopsy as described below.

**Fig. 1a:**
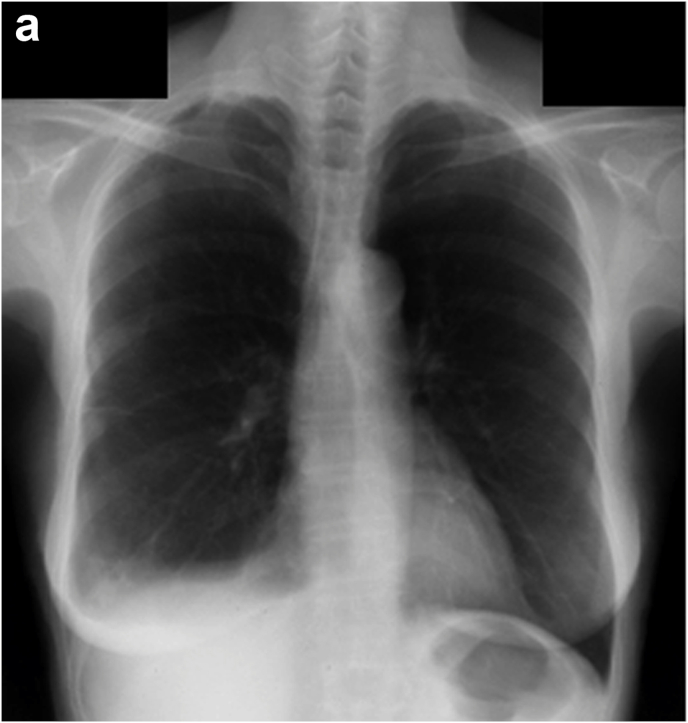
Chest radiographs July 2006 The right costophrenic angle is blunted, but no other abnormalities were noted at this point.

**Fig. 1b:**
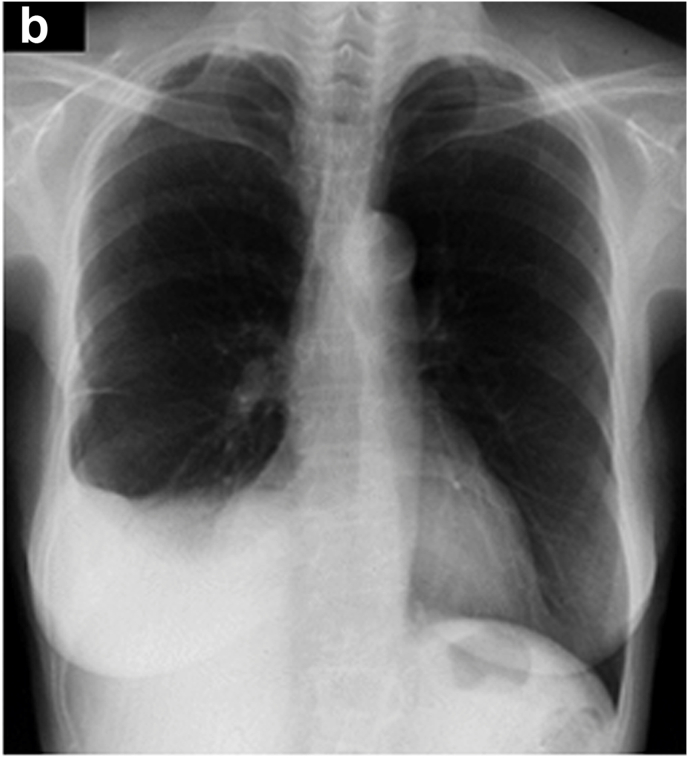
Chest radiographs Blunting of the right costophrenic angle has progressed, and a mass is observed in the thoracic wall.

**Fig. 2:**
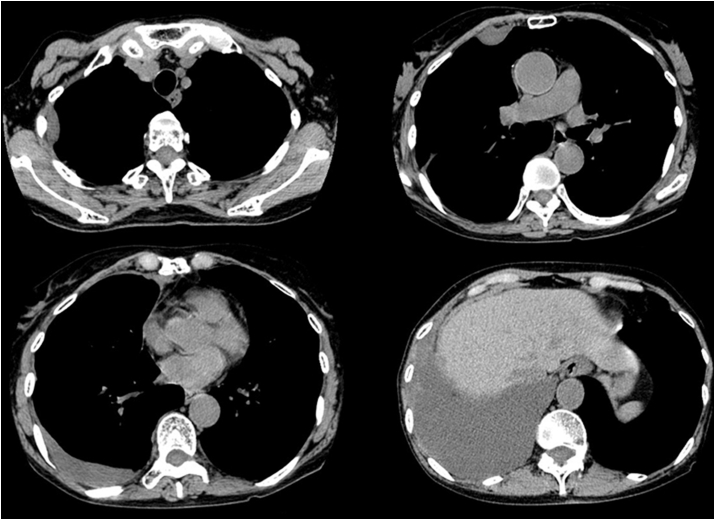
Chest plain CT scans at the first examination by the previous hospital (July 2007) A small amount of pleural effusion is present on the right side. Pleural masses are noted in the right anterior mediastinum and anterior and lateral thoracic regions, and irregular pleural thickening extends from the right mediastinum to the anterior thoracic wall.

Therefore, the clinician recommended anticancer drug treatment. However, as the patient did not wish for aggressive treatment, she was followed-up without treatment.

**Fig. 3:**
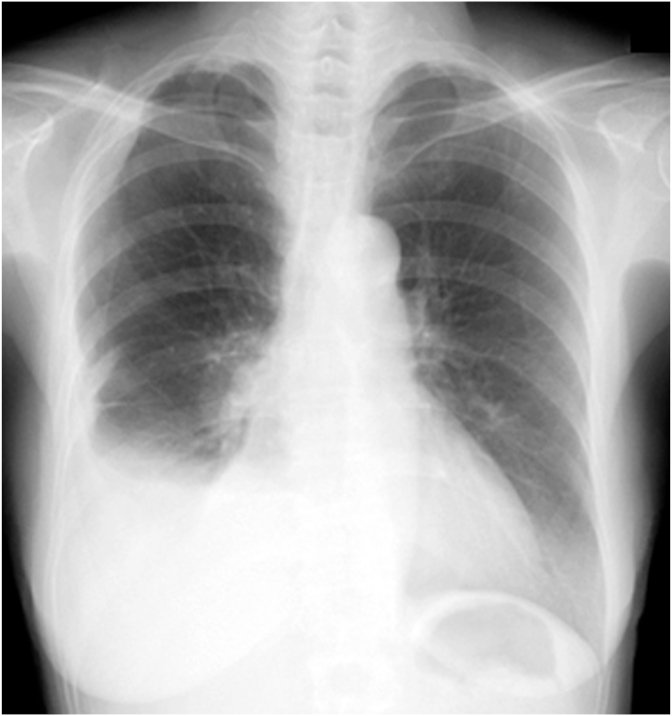
Chest radiographs taken at in April 2008. Masses were observed in the area corresponding to the peripheries of the right lower lung field and the right lateral chest wall.

Fig. 3 shows a plain chest radiograph taken in April 2008. Masses were observed in the area corresponding to the right lower lung field's peripheries and the right lateral chest wall. Dyspnea appeared in the middle of May 2010, and the patient was admitted to Toyama Rosai Hospital in late May.

### 1.2 Physical findings on admission

The patient had clear consciousness, a blood pressure of 110/42 mmHg, a regular heart rate of 102 bpm, a body temperature of 36.8 °C, and a respiratory rate of 32/min. Hypoxemia with an arterial blood oxygen tension of 67.1 Torr under inhalation of room air was noted, and the alveolar-arterial oxygen gradient was widened to 36.0 Torr. Mild anemia was noted in the palpebral conjunctiva. Breath sounds were weakened in the right lung region on chest auscultation.

### 1.3 Laboratory findings on admission

As shown in Table 1, blood tests on admission indicated anemia with a hemoglobin level of 9.9 g/dL, but the platelet count was increased at 53.0 × 10^4^/μL. CRP (8.1 mg/dL) and fibrinogen (600 mg/dL) were high, and the ESR was increased. The concentrations of tumor markers CA125 (147 μ/mL) and TPA (96 μ/mL) were elevated. The CEA, SCC antigen, and ProGRP levels were normal. The serum ERC/mesothelin level [3] was elevated at 35.5 ng/mL (cut-off value: 5–10 ng/mL) [3].

**Table 1:**
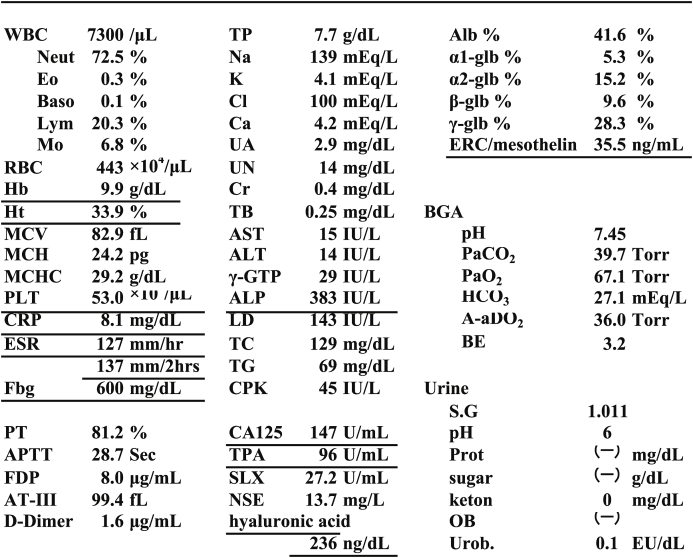
Laboratory findings on admission

### 1.4 Clinical course after admission

**Fig. 4:**
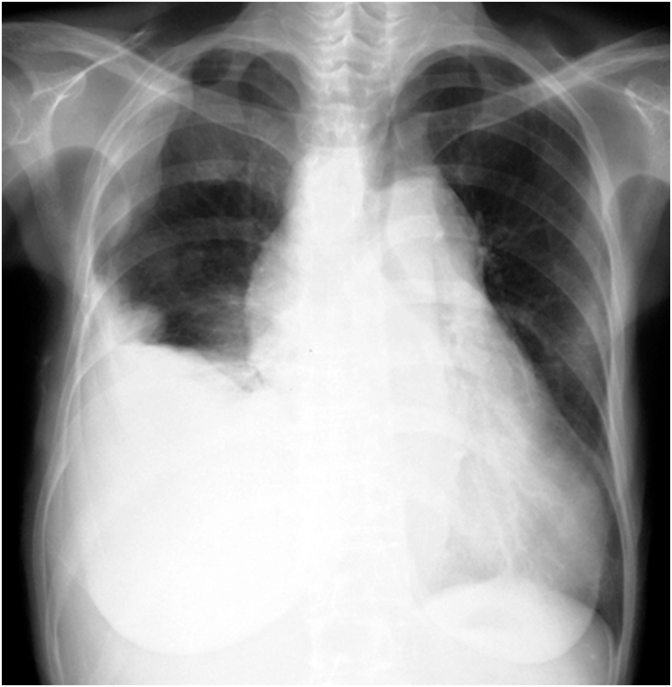
Chest radiographs taken on admission to Toyama Rosai Hospital (May 2010) The inside of the right thorax was mostly occupied by multiple masses, and the mediastinum is displaced to the left.

**Fig. 5:**
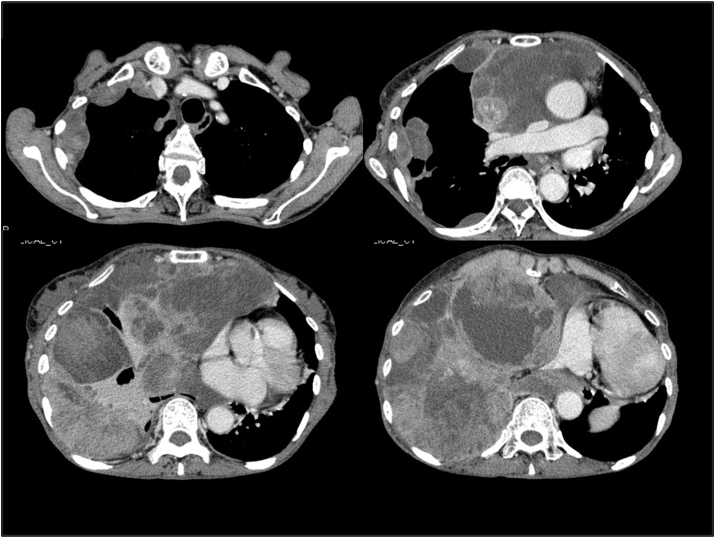
Contrast-enhanced CT scans of the chest (May 2010) The right thoracic cavity is filled with multiple masses of various sizes, and part of the masses spread to, and compressed, the left mediastinum. The interior of more than half these masses was extremely hypodense.

Fig. 4 shows plain chest radiographs, and Fig. 5 shows contrast-enhanced CT scans of the chest, taken in May 2010, when the patient was first admitted. In addition to many giant tumor-like shadows in the right lower thoracic cavity, shadows of multiple masses extending along the pleura were noted in the right upper thoracic region. Part of the interior of some masses was hypodense. A giant mass was also pointed out in the mediastinum, and the heart was markedly displaced to the left. No clear abnormal shadows suggesting metastasis or infiltration were observed in the lungs. ^18^F-FDG-PET/CT images were obtained in the same period. No apparent FDG accumulation was noted in areas other than the chest. While contrast-enhanced MRI of the head was also performed, there were no abnormal findings. Thus no extrathoracic distant metastasis was detected.

Therefore, we judged that no aggressive intervention or treatment was necessary at this point and temporarily discharged the patient in June 2010 after introducing opioid administration and home oxygen therapy.

However, the patient was re-admitted in October 2010 due to worsening of dyspnea. After that, the masses in the right thoracic cavity further enlarged.

The patient died at the end of January 2011 due to respiratory insufficiency. We, fortunately, had an opportunity to perform an autopsy. The patient was asymptomatic from 2005. This time is considered to be the time of onset from the radiographs. The diagnosis was made by needle biopsy in August 2007. The patient was observed without treatment until about April 2008. The disease is considered to have progressed slowly during this period but rapidly after that.

### 1.5 Pathological findings by autopsy

**Fig. 6:**
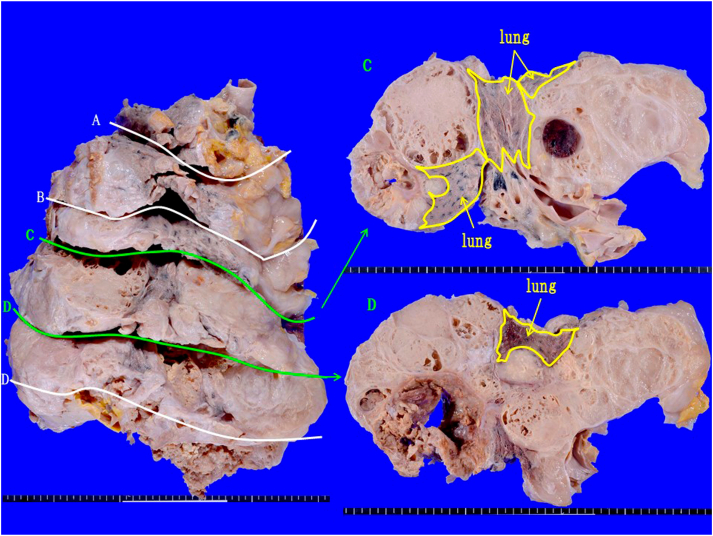
Macroscopic views of an autopsy sample A whole picture of a giant mass occupying the right lower thoracic cavity. It was a gelatinous, lobulated, and grayish white tumor showing displacive proliferation and enveloping the lung.

The lesion located in the lower right thoracic cavity was a large lobulated mass that extended widely around the right lung and squeezed it. Necrosis/degeneration was notable in some areas, but translucent collagenous grayish-white areas were predominant. These areas corresponded to the non-enhancing and highly radiolucent nodules in the mass. Thease nodules are among the multiple mass-like shadows observed in contrast-enhanced chest CT scans(Fig. 5). They were identified with the part of the tumor rich in hyaluronic acid myxoid stroma (Fig. 6).

**Fig. 7:**
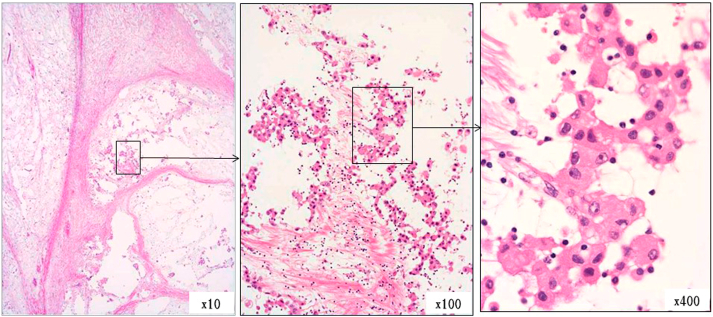
HE-stained autopsy sample. A multinodular lesion rich in myxoid stroma segmented into lobules by fiber bundles. Epithelioid tumor cells floating in myxoid stroma are observed.

On histological examination, the lesion showed a lobulated structure segmented by fiber bundles, and while some parts were solid, rich in cellular components, and necrosed/degenerated, many features exhibited mildly atypical epithelioid tumor cell proliferation consisting of eosinophilic cells rich in the myxoid stroma, low in density, and with a binding tendency (Fig. 7). These features differed from those of a well-differentiated papillary tumor. Infiltrative proliferation into the lungs and mediastinum was observed in some areas, but displacive proliferation was predominant. No lymph node metastasis or distant metastasis was noted.

**Fig. 8:**
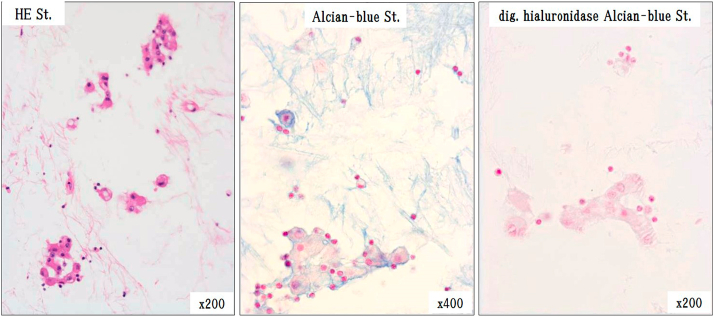
Alcian blue stain and hyaluronidase digestion test of autopsied tissue. Myxoid stroma was shown to consist of hyaluronic acid, because it is stained with Alcian blue and digested by hyaluronidase. . (For interpretation of the references to colour in this figure legend, the reader is referred to the Web version of this article.)

Myxoid stroma and tumor cells were stained with Alcian Blue and consisted of hyaluronic acid mucus digested by hyaluronidase (Fig. 8). Immunohistochemically, the lesion was positive for Calretinin, D2-40, WT1. Immunohistochemical staining was positive for HEG1(focal) in a small number of tumor cells and EMA (membrane, focal), while was negative for CEA, TTF-1 and desmin.

**Fig. 9:**
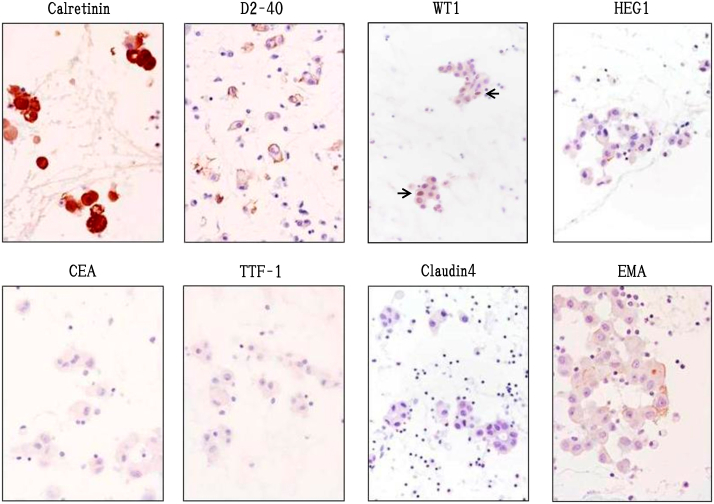
Immunohistochemical staining. Tumor cells were positive for calretinin, D2-40, WT1, and HEG1 (focal) and negative for CEA and TTF-1. EMA (focal) was positive in the membrane part of the cell.

The loss of BAP1 in tumor cells could not be assessed because the lymphocytes, which should be the internal positive controls, were not stained with BAP1.We guess that the reason for the poor staining of lymphocytes is that it has been more than eight years since the autopsy. (Fig. 9) (Table 2). The Ki-67 index, which was examined simultaneously, varied, being 12.2% in stroma-rich areas but 17.2% in stroma-deficient regions, and was higher than 3.3% determined by needle biopsy in 2007 at the previous hospital. From these findings, a diagnosis of epithelioid pleural mesothelioma (myxoid variant) was made. The number of asbestos bodies detected in the autopsied lungs was 627/g (dry lung), which did not differ from the population average.

**Table 2:** List of immunohistochemical staining results

Antibody	Reaction	Antibody	Reaction
CK (AE1/AE3)	Positive, diffuse	CEA	Negative
Calretinin	Positive, diffuse	TTF-1	Negative
D2-40	Positive, focal	Naspin A	Negative
WT1	Positive, a few	Ber-EP4	Negative
CK5/6	Positive, diffuse	MOC-31	Negative
Mesothelin	Positive, diffuse	Claudin4	Negative
HEG1	positive, focal	Desmin	Negative
EMA	Positive.(membrane, focal)	MTAP	No defect
Ki-67 index	Stroma-rich area: 12.2% Stroma-deficient area: 17.2%		
	(Ki-67 index of biopsy sample obtained in 2007: 3.3%)		

## 2 Discussion

While the findings in the chest radiographs taken on community health screening in July 2005 were considered normal, the right costophrenic angle was retrospectively found to be blunted, so pleural effusion is believed to have appeared before July 2005. Pleural effusion is reported to be observed at the onset of pleural mesothelioma in 91% of patients [4]. After two years, in July 2007, masses appeared ipsilaterally in the thoracic region with an increase in the amount of right thoracic effusion, and pleural mesothelioma was diagnosed by needle biopsy in August of the same year. Therefore, it is highly likely that pleural mesothelioma accompanied by pleural effusion had developed before the detection of right pleural effusion in July 2005 and that we observed the subsequent course of progression. While the patient was observed after that without treatment until about April 2008, she remained asymptomatic, and progression of the disease was slow. After that, however, the disease is considered to have progressed rapidly, resulting in death in late January 2011. The patient is considered to have survived for five years and six months after the onset (3 years and six months after the diagnosis) without treatment.

The lesion was definitively diagnosed as epithelioid pleural mesothelioma (myxoid variant) through detailed pathological evaluation by autopsy, and the prognosis was more favorable than usual mesothelioma.

We searched ICHUSHI (Japan centra revuo medicina) as of May 2020 for reports of pleural mesothelioma patients' long-term survival. A search was made using “pleural mesothelioma” and “long-term survival” as keywords in a period of about 30 years after 1981. Case reports with mesothelioma diagnoses made by tissue biopsy, clear histological types, non-surgical treatments, and survival for five years or longer were selected. As shown in Table 3, 7 case reports met the above conditions, and they were all-conference proceedings.

**Table 3:** Long-term survival pleural mesothelioma case search results

Case No.	Year of publication	Form of publication	Authors	Affiliation	Journal	Volume (issue)	Page	Biopsy	Histological type	Primary treatment	Survival period	Dead or alive
1	2019	Conference proceedings	Fukushima et al.	Ichinomiya Municipal Hospital	Lung Cancer	59(6)	402	Percutaneous needle biopsy	Epithelioid	CDDP + PEM, PEM, VNR	≥6 years	Alive
2	2019	Conference proceedings	Oyama et al.	Koto Hosptal	Annals of The Japanese Respiratory Society	Vol.8	361	Pleural fluid cell block	Epithelioid	Repeated drainageof pleural effusion	10 years	Dead
3	2014	Conference proceedings	Suzuki et al.	Seirei Mikatahara General Hospital	Lung Cancer	54(1)	38–39	CT-guided biopsy	Epithelioid	CDDP + GEM	5y.9m.	Dead
4	2014	Conference proceedings	Suzuki et al.	Seirei Mikatahara General Hospital	Lung Cancer	54(1)	38–39	Thoracoscopy	Epithelioid	CDDP + PEM	8y.3m.	Dead
5	2008	Conference proceedings	Yoshida et al.	Gunma University Hospital	Thermal Medicine	24(1)	17–18	Thoracoscopy	Sarcomatoid	CDDP + CPT11	5y.2m.	Alive
6	2007	Conference proceedings	Tsukada et al.	Yokosuka Kyosai Hospital	Lung Cancer	47(1)	71	Thoracoscopy Autopsy	Biphasic	Palliative therapy	9y.3m.	Dead
7	2002	Conference proceedings	Kano et al.	Kanazawa University Hospital	Lung Cancer	42(3)	233	Thoracoscopy	Epithelioid	CDDP + ADM + VP-16(a)	8 years	Alive

a Intrathoracic.

The histological type was sarcomatoid and biphasic in 1 case each and epithelioid in the remaining 5. Case 2 was 80 years old at the time of diagnosis and reportedly survived for ten years by repeated drainage of pleural effusion alone. Case 6 received palliative treatment alone, and Case 5 with sarcomatoid mesothelioma was still alive with combination chemotherapy of cisplatin + irinotecan. The histological type was mostly epithelioid, and some of the 5 cases were reported as epithelioid mesotheliomas, which may have been myxoid variants.

It has recently been proposed that epithelioid mesothelioma rich myxoid stroma is a histological characteristic associated with a favorable prognosis. Such lesions can be identified as a subtype of epithelioid mesothelioma. Shia et al. studied 19 patients with epithelioid pleural mesothelioma with pronounced myxoid stroma, in which myxoid components accounted for ≥50%, and reported that the percentage of females (47%) was higher and that the percentage of those with a history of asbestos exposure (53%) was lower than in patients with usual pleural mesothelioma [5]. Also, Alchami et al. evaluated the histological morphology and prognosis in 191 patients with pleural mesothelioma and, by subclassifying epithelioid mesotheliomas according to pronounced morphological features, reported that the prognosis was more favorable with statistical significance in the myxoid/microcystic subtype, with a median survival time of 24 months and a 2-year survival rate of 50% than the tubulopapillary, solid, micropapillary, and pleomorphic subtypes [6]. Based on the report by Shia et al., 2016 WHO classification of tumors of the pleura described the prognosis of epithelioid mesothelioma with abundant myxoid change as favorable [6]. It is necessary to recognize this subtype of epithelioid mesothelioma rich in myxoid components as a myxoid variant, and the present case supports this view. This is considered to be the first detailed case report of a myxoid variant of epithelioid pleural mesothelioma from Japan.

Shia et al. reported that the Ki-67 index was <5% in 7 and 10–30% in 5 of 19 patients [6]. In our present case, also, it was 3.3% by biopsy at the previous hospital and 17.2% even in the stroma-deficient part by autopsy, indicating that the tumor was not highly malignant.

In the present case, right pleural effusion was clearly observed on plain chest radiographs taken during a community health screening in July 2005, but the findings remained unchanged for one year. Although mass shadows were noted in the chest the next year, it took three years for subjective dyspnea to appear. When the needle biopsy was performed in 2007, neither pathologists nor clinicians knew that the mesothelioma shows the characteristic feature of the myxoid stroma. Also, clinicians did not know that mesothelioma patients with this pathology had a good prognosis. Although chest radiograms after the transfer to Toyama Rosai Hospital in 2010 confirmed the accelerated progression of the disease.

Although mass shadows were noted in the chest the next year, It took 3 years for the subjective complaint of dyspnea. When the needle biopsy was performed in 2007, neither pathologists nor clinicians knew that the mesothelioma shows the characteristic feature of the myxoid stroma. Also, clinicians did not know that mesothelioma patients with this pathology had a good prognosis. Although chest radiograms after transfer to Toyama Rosai Hospital in 2010 confirmed the accelerated progression of this disease. Neither pathologists nor clinicians knew that mesothelioma patients with this pathology had a good prognosis.

The tumor is considered to have grown slowly in the early stages but enlarged rapidly and displaced the surrounding tissues after a certain point.

When a myxoid variant of epithelioid mesothelioma has been diagnosed, it may be necessary to develop a therapeutic strategy different from the one for usual pleural mesothelioma, because the disease may progress slowly, at least in the early stages.

## 3 Conclusions

1 We reported a patient with pleural mesothelioma who survived for five years and six months without treatment.
2 Pathologically, the lesion was a myxoid variant of epithelioid mesothelioma rich in a myxoid stroma.
3 The myxoid variant of epithelioid mesothelioma is characterized as a good-prognosis subtype of epithelial mesothelioma, and the present case supports this view.
4 Patients with such pleural mesothelioma may live relatively long even without treatment, and the selection of treatment appropriate for the histological type based on an accurate diagnosis is considered necessary.

An abstract of this paper was presented at the 60th Annual Meeting of the Japanese Society of Occupational Medicine and Traumatology (November 5th-6th, 2010; Chiba City).

## Declaration of competing interest

The authors declare no conflicts of interest associated with this manuscript.
